# Understanding apoptosis in sickle cell anemia patients: Mechanisms and implications

**DOI:** 10.1097/MD.0000000000036898

**Published:** 2024-01-12

**Authors:** Emmanuel Ifeanyi Obeagu, Nwanganga Ihuoma Ubosi, Getrude Uzoma Obeagu, Simeon Ikechukwu Egba, Martin H. Bluth

**Affiliations:** a Department of Medical Laboratory Science, Kampala International University, Kampala, Uganda; b Department of Public Health Sciences, Faculty of Health Sciences, National Open University of Nigeria, Headquarters, Jabi, Abuja, Nigeria; c School of Nursing Science, Kampala International University, Kampala, Uganda; d Department of Biochemistry, Michael Okpara University of Agriculture, Umudike, Abia State, Nigeria; e Department of Pathology, Division of Blood Transfusion Medicine, Maimonides Medical Center, Brooklyn, NY, USA.

**Keywords:** apoptosis, inflammation, reactive oxygen species, sickle cell anemia, vaso-occlusive crises

## Abstract

Sickle cell anemia (SCA) is a hereditary blood disorder characterized by the presence of abnormal hemoglobin, leading to the formation of sickle-shaped red blood cells. While much research has focused on the molecular and cellular mechanisms underlying the pathophysiology of SCA, recent attention has turned to the role of apoptosis, or programmed cell death, in the disease progression. This review aims to elucidate the intricate mechanisms of apoptosis in SCA patients and explore its implications in disease severity, complications, and potential therapeutic interventions. Different research search engines such as PubMed central, Scopus, Web of Science, Google Scholar, ResearchGate, Academia Edu, etc were utilized in writing this paper. Apoptosis, a highly regulated cellular process, plays a crucial role in maintaining homeostasis by eliminating damaged or dysfunctional cells. In SCA, the imbalance between pro-apoptotic and anti-apoptotic signals contributes to increased erythrocyte apoptosis, exacerbating anemia and vaso-occlusive crises. Various factors, including oxidative stress, inflammation, and altered cell signaling pathways, converge to modulate the apoptotic response in SCA. Furthermore, the interaction between apoptotic cells and the vascular endothelium contributes to endothelial dysfunction, promoting the pathogenesis of vasculopathy and organ damage seen in SCA patients. In conclusion, unraveling the complexities of apoptosis in SCA provides valuable insights into the disease pathophysiology and offers novel avenues for therapeutic interventions.

## 1. Introduction

Sickle cell anemia (SCA) is a hereditary blood disorder that has long been recognized for its hallmark feature—the misshapen, crescent-like red blood cells responsible for vaso-occlusive crises, severe pain, and multiple organ complications.^[1]^ This genetic condition arises from a single point mutation in the beta-globin gene, leading to the production of abnormal hemoglobin known as hemoglobin S (HbS).^[2]^ The polymerization of deoxygenated HbS causes red blood cells to become rigid and distorted, leading to their entrapment in blood vessels, decreased oxygen delivery, and the clinical manifestations associated with SCA.^[3–5]^

While the distorted morphology of red blood cells is central to the pathology of SCA, it is increasingly evident that other molecular processes contribute to the complexity of the disease.^[6]^ Among these processes, apoptosis, the regulated form of programmed cell death, has emerged as a topic of growing interest in SCA research.^[7]^ This newfound attention to apoptosis reflects the realization that the pathophysiology of SCA extends beyond the structural abnormality of red blood cells and involves intricate molecular mechanisms.^[8]^ Apoptosis, which plays a fundamental role in maintaining cellular homeostasis and tissue development, has now been identified as an integral part of the pathophysiological landscape of SCA.^[9]^ It is a tightly regulated process, involving a cascade of molecular signals and cellular events, leading to the programmed self-destruction of a cell. Dysregulation of apoptosis is associated with various diseases, including cancer, neurodegenerative disorders, and autoimmune conditions.^[10]^ In the context of SCA, apoptosis introduces an intriguing dimension to the understanding of the disease.

This paper endeavors to explore the multifaceted role of apoptosis in SCA. By unraveling the molecular mechanisms driving programmed cell death, the implications for disease progression, and the potential therapeutic interventions, we aim to provide a comprehensive and updated perspective on this critical aspect of SCA pathophysiology. Understanding apoptosis in SCA holds the promise of not only shedding light on the complexities of the disease but also offering new avenues for therapeutic strategies that can improve the quality of life and overall outcomes for individuals living with SCA.

## 2. Methods

Different research search engines such as PubMed central, Scopus, Web of Science, Google Scholar, ResearchGate, Academia Edu, etc were utilized in writing this paper.

### 2.1. Ethical approval

Not applicable as this a narrative review paper.

### 2.2. Apoptosis

Apoptosis, often referred to as programmed cell death, is a fundamental biological process that plays a crucial role in maintaining tissue homeostasis, development, and the elimination of damaged or unwanted cells.^[11]^ It is a highly regulated and controlled mechanism of cell suicide, distinct from necrosis, which is uncontrolled cell death due to injury or external factors. Apoptosis can be initiated by various internal and external signals, such as DNA damage, cell stress, growth factor withdrawal, or immune responses.^[12]^ These signals activate specific pathways that trigger the apoptotic process. Apoptotic cells undergo characteristic morphological changes, including cell shrinkage, chromatin condensation (resulting in pyknotic nuclei), membrane blebbing, and the formation of apoptotic bodies, which are small, membrane-bound vesicles containing cellular components.^[13]^ DNA fragmentation is a hallmark of apoptosis. Endonucleases cleave the DNA into small fragments, creating a ladder-like pattern when analyzed through gel electrophoresis. Apoptosis is regulated by a complex network of genes and proteins.^[14]^ Key players include caspases, which are proteases that cleave specific cellular substrates, and members of the Bcl-2 protein family, which control the mitochondrial pathway of apoptosis.^[15]^

In a controlled process, neighboring cells or phagocytes recognize and engulf the apoptotic bodies, preventing the release of cellular contents and inflammation.^[16]^ Apoptosis is essential during embryonic development to remove unnecessary or excess cells, shape tissues and organs, and create the appropriate structure.^[17]^ It is also critical in tissue remodeling and maintenance throughout life. Apoptosis plays a role in the immune system by eliminating damaged or infected cells and is involved in maintaining immune tolerance to self-antigens.^[18]^ Apoptosis acts as a defense mechanism against the development of cancer by eliminating cells with DNA damage or genetic mutations that could lead to malignancy.^[11]^ Dysregulation of apoptosis is associated with various diseases. Insufficient apoptosis can lead to cancer, autoimmune diseases, and neurodegenerative disorders, while excessive apoptosis is implicated in degenerative conditions and tissue damage.^[19]^ Understanding apoptosis has led to the development of therapies for diseases such as cancer, where the goal is to induce apoptosis in malignant cells. Apoptosis is a finely tuned process that balances cell survival and cell death, contributing to the maintenance of tissue and organismal homeostasis.^[20]^ It remains a subject of extensive research in various fields, including oncology, immunology, and developmental biology, with the aim of a better understanding its regulation and harnessing its potential for therapeutic purposes.

### 2.3. Mechanisms of apoptosis

**Figure 1.**
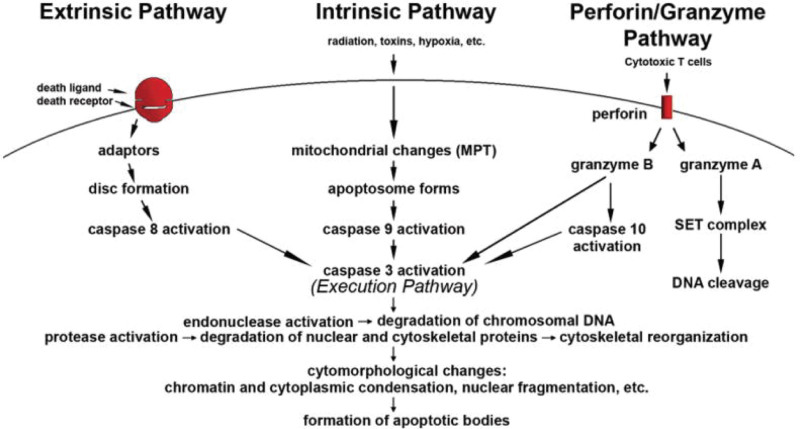
Schematic representation of apoptotic events.

Figure 1 shows schematic representation of apoptotic events.^[21]^ Apoptosis, also known as programmed cell death, is a highly regulated process crucial for the development, maintenance, and elimination of cells in multicellular organisms. It serves various purposes, including embryonic development, tissue homeostasis, and elimination of damaged or potentially harmful cells. Apoptosis can be initiated through various internal or external signals, such as DNA damage, cell stress, or signaling from other cells. These signals activate specific pathways that trigger apoptosis.^[21]^ This pathway is initiated by intracellular signals, such as DNA damage or cellular stress. It involves the permeabilization of the outer mitochondrial membrane, leading to the release of pro-apoptotic proteins like cytochrome c. Cytochrome c, along with other proteins, forms the apoptosome, which activates caspase enzymes. The extrinsic pathway is activated by external signals binding to death receptors on the cell surface. Ligands like Fas ligand or tumor necrosis factor bind to their respective death receptors, leading to the formation of the death-inducing signaling complex. This complex activates caspase enzymes directly, initiating apoptosis. Caspases are key mediators of apoptosis. They exist as inactive proenzymes and are activated through proteolytic cleavage. Initiator caspases (e.g., Caspase-8, -9) are activated early in the apoptotic process, which then activate executioner caspases (e.g., Caspase-3, -6, -7), leading to the cascade of events that result in cell death. Once activated, executioner caspases cleave various cellular substrates, leading to characteristic morphological changes in the cell, including DNA fragmentation, cytoskeletal breakdown, nuclear condensation, and membrane blebbing. Following the execution phase, the cell undergoes changes that enable its recognition and engulfment by neighboring cells or phagocytes. This prevents the release of potentially harmful cellular contents into the surrounding tissue.^[21]^ Apoptosis is tightly regulated by various factors, including anti-apoptotic proteins (e.g., Bcl-2 family), pro-apoptotic proteins (e.g., Bax, Bak), and inhibitors of apoptosis proteins. These proteins interact to maintain a balance between cell survival and cell death. The Bcl-2 family of proteins plays a crucial role in regulating the intrinsic pathway by controlling mitochondrial outer membrane permeabilization. Bcl-2 family members either promote or inhibit mitochondrial outer membrane permeabilization, thereby regulating the release of pro-apoptotic factors from mitochondria.

### 2.4. Apoptosis in SCA

Apoptosis, the programmed cell death process, has gained recognition for its significant role in SCA.^[22]^ While the hallmark feature of SCA is the presence of abnormal hemoglobin (HbS) leading to the characteristic sickle-shaped red blood cells, recent research has illuminated the role of apoptosis in the pathophysiology of the disease.^[23]^ The rigidity and fragility of sickle red blood cells make them more susceptible to undergoing apoptosis. During vaso-occlusive crises, when these cells become trapped in small blood vessels, they endure mechanical stress and oxidative damage.^[24]^ This stress can activate apoptotic pathways in sickle red blood cells, contributing to their destruction and leading to hemolysis.^[25]^ Oxidative stress is a prominent feature of SCA, driven by the polymerization of deoxygenated HbS.^[26]^ As sickle red blood cells are exposed to low oxygen levels in the circulation, they experience oxidative damage.^[27]^ The resulting oxidative stress can trigger apoptosis pathways, including the release of reactive oxygen species and the activation of pro-apoptotic proteins.^[28]^

The mitochondria play a central role in apoptosis.^[29]^ In SCA, mitochondrial dysfunction is observed in sickle red blood cells, potentially leading to the release of cytochrome c and the activation of caspases, which are central players in the apoptotic cascade.^[30]^ Apoptotic sickle red blood cells release damage-associated molecular patterns (DAMP), which are molecules that signal tissue damage and inflammation.^[31]^ DAMPs can contribute to the endothelial dysfunction observed in SCA and trigger inflammatory responses.^[32]^ The apoptotic sickle red blood cells may expose phosphatidylserine on their surface. This “consumption” signal can promote their adherence to the vascular endothelium, further contributing to vaso-occlusive crises.^[33]^ Apoptosis in SCA is implicated in the pathophysiology of acute chest syndrome, one of the life-threatening complications of the disease.^[34]^ The accumulation of apoptotic cells in the lung microvasculature can contribute to inflammation and vascular occlusion.^[35]^ Understanding the role of apoptosis in SCA offers potential therapeutic avenues. Strategies aimed at reducing the susceptibility of sickle red blood cells to apoptosis or mitigating the inflammatory response to apoptotic cells could hold promise for managing the disease. However, it important to note that the role of apoptosis in SCA is complex, with both protective and detrimental aspects. Balancing these factors is a subject of ongoing research and holds potential for improving the clinical management of this challenging blood disorder.

### 2.5. Implications of apoptosis for SCA progression

The implications of apoptosis for SCA progression are multifaceted, contributing to the overall clinical picture and complications associated with the disease.^[36]^ Apoptosis of sickle red blood cells leads to their premature destruction, a process known hemolysis.^[37]^ This results in anemia, a hallmark of SCA, characterized by reduced oxygen-carrying capacity and symptoms such as fatigue, weakness, and pallor. Apoptosis can trigger the exposure of phosphatidylserine on the surface of sickle red blood cells, promoting their adherence to the vascular endothelium. This increased adherence contributes to vaso-occlusive crises, one of the most painful and debilitating aspects of SCA, in which blood vessels become blocked by aggregates of sickled cells, leading to ischemia, pain, and tissue damage.^[38]^

The presence of DAMPs, which are released by apoptotic cells, can lead to endothelial dysfunction, impairing the normal function of the blood vessel lining and increasing the risk of complications like acute chest syndrome and pulmonary hypertension. Apoptotic sickle red blood cells and DAMPs trigger an inflammatory response that can further exacerbate vaso-occlusion and tissue damage.^[39]^ This inflammatory component of SCA progression can lead to complications in multiple organ systems. The accumulation of apoptotic cells in the lung microvasculature is associated with the development of acute chest syndrome, a potentially life-threatening complication of SCA.^[23]^ This syndrome is characterized by lung inflammation, hypoxia, and chest pain, and can lead to severe respiratory distress. The pro-inflammatory and pro-coagulant environment created by apoptotic cells and DAMPs can promote thrombosis (blood clot formation) in SCA patients, leading to further vascular complications and tissue damage.^[39]^

Over time, the cumulative impact of apoptosis and related processes can result in organ damage. This damage may manifest in the form of stroke, kidney dysfunction, liver complications, and other SCA-related organ problems. The ongoing presence of apoptosis contributes to the chronicity of SCA, with patients experiencing recurrent vaso-occlusive crises, inflammation, and tissue damage.^[40]^ Chronic complications may reduce the quality of life and life expectancy for individuals with SCA.^[41]^ Understanding the implications of apoptosis in SCA progression has significant therapeutic implications. Research into therapies that target apoptotic pathways or reduce the susceptibility of sickle red blood cells to apoptosis may offer novel treatment options for managing the disease and its complications. Apoptosis plays a multifaceted role in the progression of SCA.^[42]^ While apoptosis contributes to the hemolysis, vaso-occlusion, inflammation, and complications associated with the disease, it also holds potential as a therapeutic target.^[43]^ A deeper understanding of the implications of apoptosis in SCA is crucial for developing strategies to mitigate its detrimental effects and improve the clinical management of this complex blood disorder.

### 2.6. Therapeutic implications of apoptosis in SCA

The therapeutic implications of apoptosis in SCA are an emerging area of research with the potential to influence the management and treatment of this challenging disease.^[44]^ Targeting the specific apoptotic pathways activated in sickle red blood cells could potentially reduce the extent of cell destruction.^[45]^ Developing drugs or interventions that inhibit these pathways may help prolong the lifespan of sickle red blood cells and decrease the degree of anemia.^[46]^ Given that oxidative stress contributes to apoptosis in SCA, antioxidants may hold therapeutic potential. Antioxidant compounds such as N-acetylcysteine and vitamin E have been investigated for their ability to reduce oxidative damage in sickle red blood cells and potentially inhibit apoptotic pathways.^[47]^ Increased levels of fetal hemoglobin are associated with milder SCA symptoms.^[48]^ Therapies that induce fetal hemoglobin production, such as hydroxyurea, can help mitigate the effects of apoptosis by reducing the proportion of sickled red blood cells in the circulation.^[49]^ Targeting the inflammatory response triggered by apoptotic cells and DAMPs may reduce the incidence and severity of vaso-occlusive crises.^[39]^

Anti-inflammatory agents, including non-steroidal anti-inflammatory drugs or anti-cytokine therapies, could be explored in this context. Inhibiting the adhesion of sickle red blood cells to the vascular endothelium is a potential strategy to reduce vaso-occlusion.^[50]^ Therapies that target cell adhesion molecules, such as P-selectin or integrins, may be beneficial.^[51]^ Ongoing research aims to identify new pharmacological agents specifically designed to target apoptotic pathways in sickle red blood cells. These agents could work by modulating key proteins or signaling pathways involved in apoptosis. Stem cell and gene therapy approaches offer the potential to provide a long-lasting or even curative solution for SCA.^[52]^ These therapies may involve gene editing or the transplantation of hematopoietic stem cells to produce healthy, non-sickling red blood cells, reducing the overall burden of apoptotic cells in the circulation. As our understanding of the genetic and molecular basis of SCA improves, the potential for individualized or precision medicine approaches increases. Tailoring treatment strategies to the specific genetic and molecular characteristics of each patient disease may optimize outcomes. Recognizing the implications of apoptosis in SCA progression underscores the importance of early intervention. Diagnosing and treating the disease in its early stages may help prevent or reduce complications associated with apoptosis.^[53]^ Many of these therapeutic strategies are under investigation in clinical trials. Participation in such trials can provide access to cutting-edge treatments and contribute to the development of effective therapies for SCA.^[54]^ The therapeutic implications of apoptosis in SCA offer potential avenues to improve the management of the disease and reduce its complications.^[55]^ Research efforts are ongoing to explore these therapeutic strategies, with the goal of enhancing the quality of life and long-term outcomes for individuals living with SCA.

### 2.7. Future directions

Future directions in understanding apoptosis in SCA patients involve several potential avenues of research and clinical exploration. Research focusing on identifying specific apoptotic pathways that can be modulated or targeted for therapeutic intervention. This might involve investigating drugs or interventions that can inhibit or regulate these pathways to prevent excessive cell death in SCA.^[56–60]^ Advancements in precision medicine may allow for more tailored treatments based on an individual unique genetic and molecular characteristics. Understanding the specific apoptotic mechanisms in each patient could lead to personalized therapeutic strategies. Continued exploration of gene editing techniques, such as CRISPR-Cas9, for modifying genes involved in apoptosis or modifying the genetic mutation responsible for SCA. Gene therapy approaches aiming to correct or modify the genetic defects contributing to SCA might have implications for controlling apoptosis in these patients.^[61,62]^ Identification of biomarkers associated with apoptotic pathways in SCA could aid in disease monitoring, prognosis, and treatment response assessment. These biomarkers might provide insights into disease progression and help in the development of novel diagnostic tools. As more is understood about the apoptotic mechanisms in SCA, it opens the door for conducting clinical trials to test new drugs, therapeutic strategies, or interventions targeting these specific pathways. Investigating the potential synergistic effects of combining therapies that target different aspects of SCA pathophysiology, including both the underlying mutation and apoptotic processes, to achieve better outcomes. Further research could focus on how apoptotic pathways contribute to complications associated with SCA, such as organ damage, stroke, or pulmonary complications.^[63,64]^ Understanding these mechanisms might lead to strategies for preventing or minimizing these complications. Future directions might also include initiatives to educate patients about the role of apoptosis in SCA, potential treatment developments, and lifestyle modifications that could complement medical interventions. Advancements in understanding the intricate mechanisms of apoptosis in SCA hold promise for the development of novel therapeutic approaches and improved management strategies to mitigate the impact of the disease on affected individuals. These future directions signify ongoing efforts to translate research findings into clinical applications for the benefit of patients with SCA.

## 3. Conclusion

SCA, a hereditary blood disorder characterized by the presence of abnormal hemoglobin, has long been recognized for its vaso-occlusive crises and associated clinical complications. However, the understanding of the disease has evolved, revealing the intricate role of apoptosis in its pathophysiology. Apoptosis, the highly regulated process of programmed cell death, emerges as a significant player in SCA, impacting the clinical course and outcomes of affected individuals. The implications of apoptosis in SCA are far-reaching and multifaceted. Apoptosis contributes to hemolysis, vaso-occlusion, inflammation, and chronic complications, affecting multiple organ systems. Understanding apoptosis role provides not only a deeper comprehension of SCA complexities but also offers new avenues for therapeutic strategies. Potential interventions, including the inhibition of apoptotic pathways, antioxidant therapies, and anti-inflammatory approaches, hold promise for improving the clinical management of the disease.

## Author contributions

**Conceptualization:** Emmanuel Ifeanyi Obeagu.

**Methodology:** Emmanuel Ifeanyi Obeagu, Getrude Uzoma Obeagu, Martin H. Bluth.

**Supervision:** Emmanuel Ifeanyi Obeagu, Martin H. Bluth.

**Validation:** Emmanuel Ifeanyi Obeagu.

**Visualization:** Emmanuel Ifeanyi Obeagu, Nwanganga Ihuoma Ubosi, Getrude Uzoma Obeagu.

**Writing—original draft:** Emmanuel Ifeanyi Obeagu, Nwanganga Ihuoma Ubosi, Getrude Uzoma Obeagu, Simeon Ikechukwu Egba, Martin H. Bluth.

**Writing—review & editing:** Emmanuel Ifeanyi Obeagu, Nwanganga Ihuoma Ubosi, Getrude Uzoma Obeagu, Simeon Ikechukwu Egba, Martin H. Bluth.
